# Esculetin Inhibits Cancer Cell Glycolysis by Binding Tumor PGK2, GPD2, and GPI

**DOI:** 10.3389/fphar.2020.00379

**Published:** 2020-03-27

**Authors:** Song-Tao Wu, Bo Liu, Zhong-Zhu Ai, Zong-Chao Hong, Peng-Tao You, He-Zhen Wu, Yan-Fang Yang

**Affiliations:** ^1^Faculty of Pharmacy, Hubei University of Chinese Medicine, Wuhan, China; ^2^Key Laboratory of Traditional Chinese Medicine Resources and Chemistry of Hubei Province, Hubei University of Chinese Medicine, Wuhan, China; ^3^Collaborative Innovation Center of Traditional Chinese Medicine of New Products for Geriatrics Hubei Province, Hubei University of Chinese Medicine, Wuhan, China; ^4^Department of Biochemistry and Molecular Biology, Beijing Normal University, Beijing Key Laboratory, Beijing, China

**Keywords:** glycolysis, esculetin, proteomics, microscale thermophoresis, transcriptome, protein inhibitor, GPD2/GPI

## Abstract

Glycolysis can improve the tolerance of tissue cells to hypoxia, and its intermediates provide raw materials for the synthesis and metabolism of the tumor cells. If it can inhibit the activity of glycolysis-related enzymes and control the energy metabolism of tumor, it can be targeted for the treatment of malignant tumor. The target proteins phosphoglycerate kinase 2 (PGK2), glycerol-3-phosphate dehydrogenase (GPD2), and glucose-6-phosphate isomerase (GPI) were screened by combining transcriptome, proteomics, and reverse docking. We detected the binding constant of the active compound using microscale thermophoresis (MST). It was found that esculetin bound well with three potential target proteins. Esculetin significantly inhibited the rate of glycolysis, manifested by differences of cellular lactate production and glucose consumption in HepG2 cells with or without esculetin. It was found that GPD2 bound strongly to GPI, revealing the direct interaction between the two glycolysis-related proteins. Animal tests have further demonstrated that esculetin may have anticancer effects by affecting the activity of PGK2, GPD2, and GPI. The results of this study demonstrated that esculetin can affect the glucose metabolism by binding to glycolytic proteins, thus playing an anti-tumor role, and these proteins which have direct interactions are potential novel targets for tumor treatment by esculetin.

## Introduction

Through the analysis of liver cancer tissues, researchers found many characteristic metabolic markers, revealing the Warburg effect in liver cancer (Huang et al., 2013), that is, the abnormal metabolism of tumors was mainly manifested by the increase of glycolysis level, the inhibition of Tricarboxylic acid (TCA) cycle, and the significant down-regulation of dehydrogenase activity related to gluconeogenesis and fatty acid metabolism (Bensaad and Harris, 2014). Various metabolites produced during glycolysis, such as the PGK2 and GPI are involved in the synthesis of nucleic acids and fatty acids for cancer cells (Akram, 2013; Ganapathy-Kanniappan and Geschwind, 2013). At the same time, the exosomes of cancer cells promote the initiation of gluconeogenesis and detoxification of tumor-related fibroblasts, converting the metabolites of cancer cells, such as lactate and ammonia, into acetic acid and amino acids for cell proliferation (Spinelli et al., 2017; Dasgupta et al., 2018; Yan et al., 2018). Therefore, a growing number of studies have focused on the specific metabolic pattern of tumor cells, that is, inhibiting the proliferation of tumor cells and inducing apoptosis by inhibiting the glycolysis process of tumors (Talekar et al., 2014; Li et al., 2015). Many experiments show that the PGK2, GPD2), and GPI are key enzymes of glycolysis, and in promoting tumor cells play an important role in the process of glycolysis. These enzymes may be potential biomarkers and target tumor diagnosis and treatment.

Phosphoglycerate kinase 2 (PGK2) is an important enzyme in the glycolysis pathway, which can catalyze the conversion of glycerol-1, 3-diphosphate into 3-phosphoglycerate, and simultaneously produce ATP (Wu et al., 1997). PGK2 can affect the replication and repair of DNA in mammalian nuclei (Vishwanatha et al., 1992; Popanda et al., 1998). PGK2 expression is regulated by oxygen tension, and its increased expression level often reflects faster tumor growth and stronger anaerobic growth habit (Semenza et al., 1994; Semenza, 1999). For example, in tumor tissues of patients with lung adenocarcinoma, high PGK2 expression shows worse prognosis of patients (Chen et al., 2003). It has been found that human ovarian cancer cell line SW626[TR] shows significant resistance to the anticancer drug taxol when PGK2 is highly expressed (Duan et al., 2002).

GP shuttles between mitochondria and cytoplasmic processes. It depends on the high expression of GPD2. To date, three possible metabolic effects have been proposed: “(I) the reoxidation of cytoplasmic Nicotinamide adenine dinucleotide (NADH) in glycolytic cells; (II) the cytosolic NADH oxidation process may have potential implications for heat generation and/or metabolic efficiency; and (III) regulation of cytoplasmic glycerol 3-phosphate (G3P) is a metabolite associated with glycolysis, lipogenesis, and OXPHOS” (Mráček et al., 2013).

Glucose phosphate isomerase (GPI) can catalyze the exchange of the isomers of glucose 6-phosphate (Rose, 1975). Glycolysis and gluconeogenesis are indirectly affected by this reaction. Just like nerve interleukin, maturation factor, and autocrine motor factor, cell proliferation and metastasis can be promoted by GPI (Gurney et al., 1986a; Gurney et al., 1986b; Watanabe et al., 1996).

*Fraxinus chinensis* subsp. *rhynchophylla* (Hance) A.E. Murray is a widely-distributed resource in China. Esculetin is the active compound of it and has been shown to inhibit cell proliferation in human colon cancer through the Ras/ERK1/2 pathway (Park et al., 2011). It was reported that esculetin induced apoptosis of U937 cells (Park et al., 2008). In addition, HepG2 cells have been shown to be sensitive to the effects of paclitaxel, when using esculetin at the same time (Kuo et al., 2006). At the same time, the high dose of esculetin can regulate the PTEN/Akt signaling pathway and inhibit the phosphorylation of Akt, leading to attenuated expression of Snail and Twist to inhibit the migration of PC3 cells in prostate cancer (Turkekul et al., 2018).In this study, transcriptomics was used to identify proteins that were differentially expressed in the total protein incubated with its active compound or not. According to network pharmacology, potential protein targets were then identified. Using microscale thermophoresis (MST), it was shown that proteins had good binding to the active compound. Combined with proteomics, *in vitro* and *in vivo* experiments, these results demonstrate that esculetin could play an anti-tumor role by inhibiting the activity of glycolysis-related enzymes, among which GPD2 and GPI have a strong binding.

## Materials and Methods

### Chemical and Reagents

Esculetin (CAS:531-75-9) was purchased from National Institutes for Food and Drug Control (China) (purity ≥ 99.18%). PGK2 (ab123166), GPD2(ab153790), GPI (ab208311), PGK2 monoclonal antibody (ab183031), and GPD2 monoclonal antibody (ab182144) were purchased from Abcam (Cambridge, UK). GPI monoclonal antibody (8866S), Casepase-3 antibody (9662), BAX antibody (2772T), and Bcl-2 Rabbit mAb (3498T) was obtained from Cell Signaling Technology (Boston, USA). Bovine serum albumin (BSA) was purchased from Sigma (St. Louis, MO, USA). TruSeq™ RNA sample preparation Kit from Illumina (San Diego, CA). SuperScript double-stranded cDNA synthesis kit (Invitrogen, CA).

### Cell Culture

The HepG2 cells were inoculated into six-well plates at a concentration of 5 x 10^5^/ml. There are two groups: HepG2 cells without esculetin and HepG2 cells containing esculetin (n=3). According to the relevant literature (Wang et al., 2015), esculetin was added at 50 µM to each well and incubated for 24 h at 37°C with 5% CO_2_.

### RNA Extraction

According to the manufacturer’s instructions (Invitrogen), getting the total RNA from HepG2 cells. RNA quality was determined. Sequencing libraries were constructed using only high-quality RNA samples (OD260/280 = 1.8~2.2).

### Library Preparation, and Illumina HiSeq X Ten Sequencing

According to the TruSeq™ RNA sample preparation Kit of Illumina, we prepared the RNA-seq transcriptome library by using 5 μg of total RNA. For a short period of time, we isolated mRNA by oligo(dT) beads and RNA was fragmented through a fragment buffer. The SuperScript double-stranded cDNA synthesis kit was used to synthesize double-stranded cDNA. The 200–300 bp cDNA target fragments size on 2% low range ultra agarose were selected, and 15 PCR cycles were amplified using Phusion DNA polymerase (NEB). Illumina HiSeq X Ten (2 × 150 bp read length) was sequenced using the paired-end RNA-seq sequencing library after quantification by TBS380.

### Differential Expression Analysis and Functional Enrichment

The expression level of each transcript was calculated by the number of segments per kilobase of exon per million mapped reads (FRKM) method, in order to identify differential expression genes (DEGs) between two different samples. RSEM (http://deweylab.biostat.wisc.edu/rsem/) (Li and Dewey, 2011) was used for quantitative genetic abundances. “The R statistical software package EdgeR (Empirical analysis of Digital Gene Expression in R, http://www.bioconductor.org/packages/2.12/bioc/html/edgeR.html)” (Robinson et al., 2010) was used to assess differences. Functional enrichment was performed using gene ontology (GO) and Kyoto Encyclopedia of Genes and Genomes (KEGG). “GO functional enrichment and KEGG pathway analysis were performed by Goatools (https://github.com/tanghaibao/Goatools) and KOBAS (http://kobas.cbi.pku.edu.cn/home.do)” (Xie et al., 2011).

### Differentially Expressed Target Gene Prediction and Analysis

PharmMapper is a kind of free use of network tools, the pharmacophore mapping method is used to identify potential targets from the results of transcriptome. Using MalaCards and GeneCards for further screening. Uploaded all potential targets to the DAVID database. Using Gene Ontology and KEGG pathway to study the functions and pathways of enrichment.

### Construction of the Network

The network was built by Cytoscape 3.6.1 software. Inputted the active compound and target gene as the node, and if there was a connection between the two nodes, added an edge to show the connection.

The “network analysis” function was used to analyze the network. Based on the KEGG analysis, key targets of the selected path with the largest counts were analyzed. According to the literature and database, we obtained the proteins suitable for target analysis (Hopkins, 2007; Shao and Zhang, 2013).

### Measurement of the Metabolic Rate of Glucose and Lactate

Just like above, the HepG2 cells were treated or not treated with 0–200 µM esculetin (0.9% NaCl solution containing 1% EtOH) for 1 h, and the medium was collected. The concentrations of glucose and lactate in the culture medium were determined by using the Model680 automatic enzyme labeling instrument from BIO-RAD Company, USA. Glucose consumption and lactate production were calculated according to the difference between the concentrations at the start of the culture and the appropriate time. At the same time, in order to exclude the influence of cell viability on the experiment, we tested the cell viability under different incubation times and different dose concentrations (Chen et al., 2012).

### Detection of Esculetin Binding to Proteins

Esculetin was analyzed with the proteins (PGK2, GPD2, GPI, and the BSA for negative control) labeled with the Labeling Kit. LED power was set to 20%.

Monolith Nano Temper (NT) 115 was used for measuring molecular interactions. Based on the fluorescence intensities that were determined through MST measurements, and then Kd values calculated (van den Bogaart et al., 2012; Batoulis et al., 2016; Vinothkannan Ravichandran et al., 2018).

### Protein-Protein Interactions

The interaction between GPD2 and GPI with higher degree in network were detect by MST experiments. GPD2 (20 nM) was used with a gradient of 16 concentrations of unlabeled GPI. As before, cell lysate were collected to incubated overnight with anti-GPD2 antibody or normal mouse IgG (as a control) and protein A/G beads at 4°C. The co-immunoprecipitated proteins were detected by mass spectrometry and western blot. Then, bioinformatics analysis was carried out for the different genes between the two groups.

### *In Vivo* Antitumor Efficacy of Esculetin

Male BALB/c mice (18–22 g) were obtained from the Hubei Provincial Center for Disease Control and Prevention (Wuhan, China). All procedures have been approved by the Animal Care and Use Committee of Institute of Materia Medica, People’s Republic of China and the H22 cells commonly used in liver-cancer modeling in normal mice were selected. H22 cells (1 x 10^7^) were injected subcutaneously (s.c.) into the right flanks of the mice, according to the literature (Zhang et al., 2019). In our preliminary experiments, we found that the toxicity of esculetin *in vivo* was negligible at 50 mg/kg, so mice were divided into three groups of eight mice, and intraperitoneal treatment was performed as follows: control group (daily received physiological saline) and esculetin-treatment group (daily received 25 and 50 mg/kg esculetin for 15 d). Antitumor activity was evaluated by weight. Also, PGK2, GPD2, and GPI expression was measured using western blot. Different protein samples were loaded onto SDS-PAGE and then electrotransferred to PVDF membranes. The membrane was then sealed with skim milk and incubated with antibodies. Finally, the results were then visualized by the ECL detection system.

## Results

### Differential Expression of Cancer-Related Genes Following Esculetin Treatment

**Figure 1:**
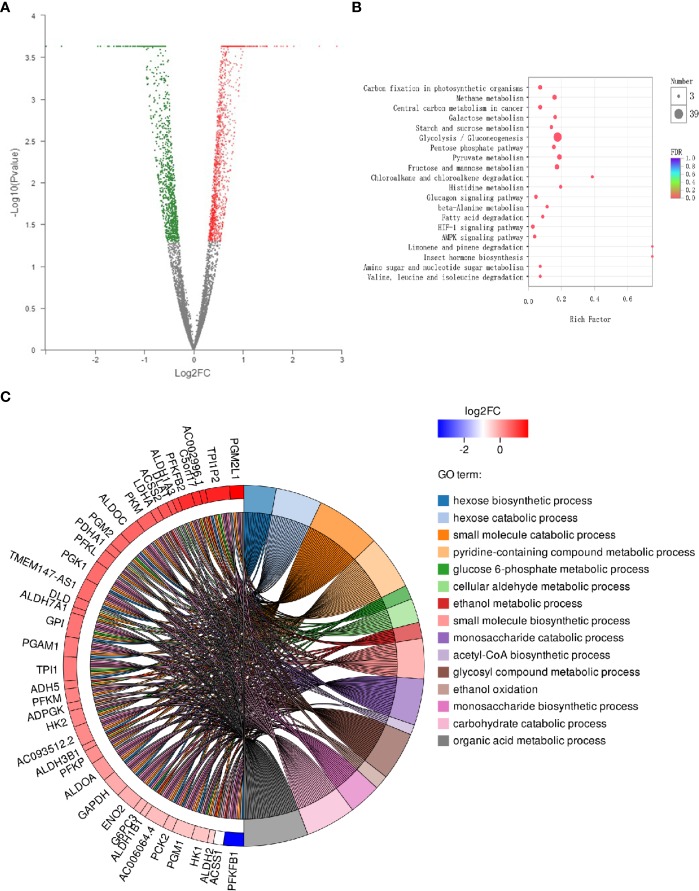
Differential expression of cancer-related genes following administration of esculetin (each group, n=3). **(A)** A volcanic map. Each dot represents a specific gene; red dots are significantly up-regulated genes, blue dots significantly down-regulated genes, and black dots represent non-significantly differentially expressed genes. The point on the left is the gene with a down-regulated expression difference, and the point on the right is the gene with an up-regulated expression difference. **(B)** KEGG enrichment analysis of cancer-related genes. The colors of the dots correspond to different Q value ranges. **(C)** GO enriched string diagrams. The larger log2FC, the larger the expression difference of up-regulated genes; the smaller log2FC is, the larger the expression difference of down-regulated genes; and the closer log2FC is to 0, the multiple of a genes differential expression was smaller. GO term information on the significant enrichment of differentially expressed genes is on the right.

The DEGs between two samples are represented in a volcanic map (**Figure 1A**). These esculetin-induced DEGs were classified into different biological process by the KEGG analysis. The top 20 clusters include the following (**Figure 1B**): glycolysis/gluconeogenesis, fatty acid degradation, glycerolipid metabolism, and fructose and mannose metabolism. The GO analysis showed that the DEGs were significantly enriched in glucose 6-phosphate metabolic processes, acetyl-CoA biosynthesis, and glycosyl compound metabolic processes (**Figure 1C**). The enriched processes of DEGs focused on the processes related to glycolysis that are specially utilized by cancer, which may be the mechanism of anticancer effect of esculetin. We further verified this in the following experiments, for more explanation of the figure, see the legend (**Table S1**).

### Target Gene Prediction and Analysis

**Figure 2:**
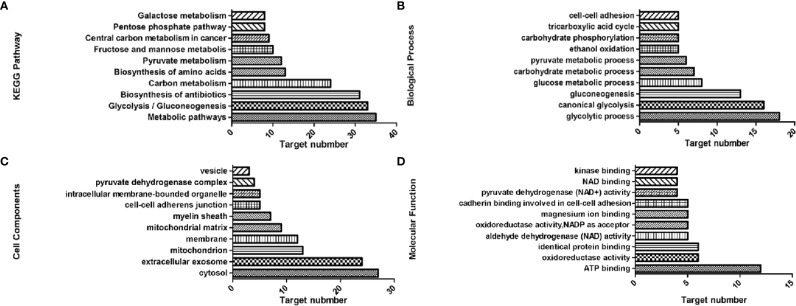
Top 10 components of the KEGG pathway and GO enrichment analyses. **(A)** KEGG pathways. **(B)** Biological process. **(C)** Cell components. **(D)** Molecular function.

KEGG analysis showed that 42 of the genes were enriched (97.7%), including 38 pathways, the target genes were significantly correlated with 33 of them (P ≤ 0.05) (**Figure 2A**). These pathways have the highest target genes involvement: Metabolic pathways (35, 81.4%), glycolysis/gluconeogenesis (33, 76.6%), carbon metabolism (24, 55.8%), pyruvate metabolism (12, 27.9%), and fructose and mannose metabolism (10, 23.3%). GO enrichment analysis showed that these genes involved in the CC (**Figure 2C**), MF (**Figure 2D**), and BP (**Figure 2B**) targets were 44 (100%). CC enrichment indicated that targets involved in those areas: cytosol (27, 62.8%), extracellular exosome (24, 55.8%), and mitochondrion (13, 30.2%). MF enrichment mainly involved target genes in the interactions: ATP binding (12, 27.9%), identical protein binding (6, 14%), and aldehyde dehydrogenase (NAD) activity (5, 11.6%). BP enrichment mostly involved targets in these processes: glycolytic process (18, 41.9%), canonical glycolysis (16, 37.2%), and gluconeogenesis (13, 30.2%).

### Network Construction

**Figure 3:**
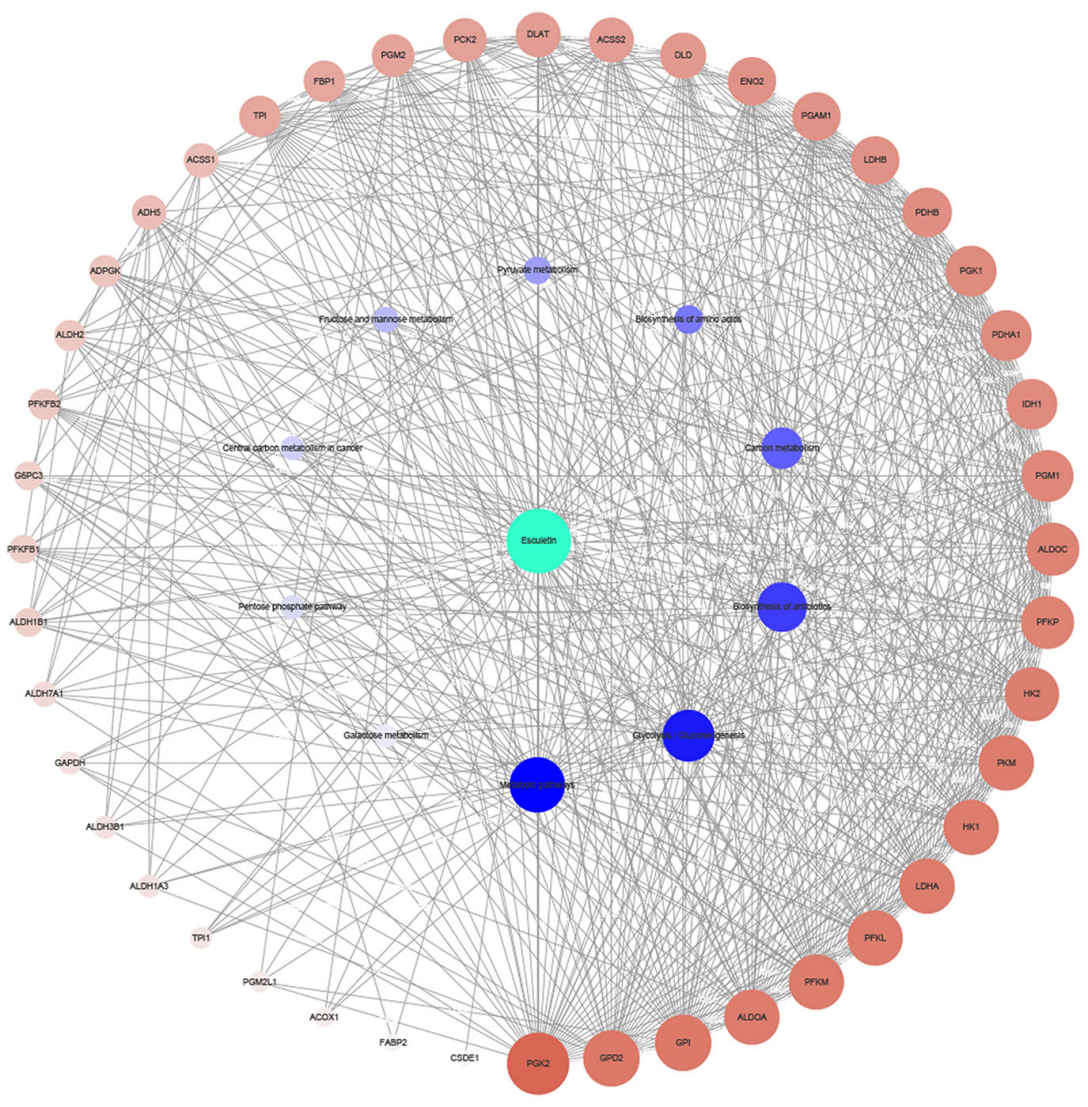
Network diagram of active component (green)/target genes (brown)/enrichment pathways (blue) constructed by Cytoscape. The bigger and darker the dot, the more correlations it has.

The target genes of the top 10 pathways and the compound were used to construct the network (**Figure 3**). The network diagram showed a synergistic effect on multiple targets for the anti-cancer effect of the esculetin. When esculetin exhibits an anti-cancer effect, GPI, GPD2, and PGK2 with a higher degree were selected for the following experiments.

### Esculetin Inhibited the Rates of Cellular Glucose Consumption and Lactate Production

**Figure 4:**
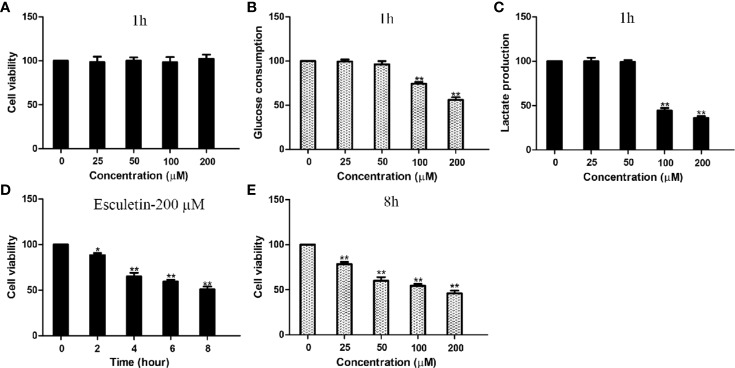
Esculetin inhibit glucose consumption and lactate production, and are toxic to cells. **(A)** The survival of HepG2 cells exposed to 0–200 µM esculetin within 1 h. **(B**, **C)** Concentration-dependent inhibition of glucose consumption and lactate production in HepG2 cells by esculetin within 1 h. **(D)** Time-dependent survival of HepG2 cells exposed to 200 µM esculetin. **(E)** Sensitivity of HepG2 cells in 8 h toward series concentration of esculetin. Data are mean ± SD (n=3), *P < 0.05, **P < 0.01.

Inhibitions of PGK2, GPD2, and GPI, were reflected by changes in glucose consumption and lactate production in the cell. Cellular glucose consumption, lactate production, and cell death were determined, after cells were treated with 0–200 µM esculetin within 1 h. The results showed that esculetin inhibited glucose consumption and lactate production in cells (**Figures 4B, C**). Considering that esculetin may kill cells and might lead to a decrease in glycolytic flux, we studied cell mortality, which were negligible for 1-h incubation (**Figure 4A**). Consistent with its inhibition on glycolytic flux, HepG2 cells were toxic under given experimental condition (**Figures 4D, E**).

### Protein Binding to Small Molecules

**Figure 5:**
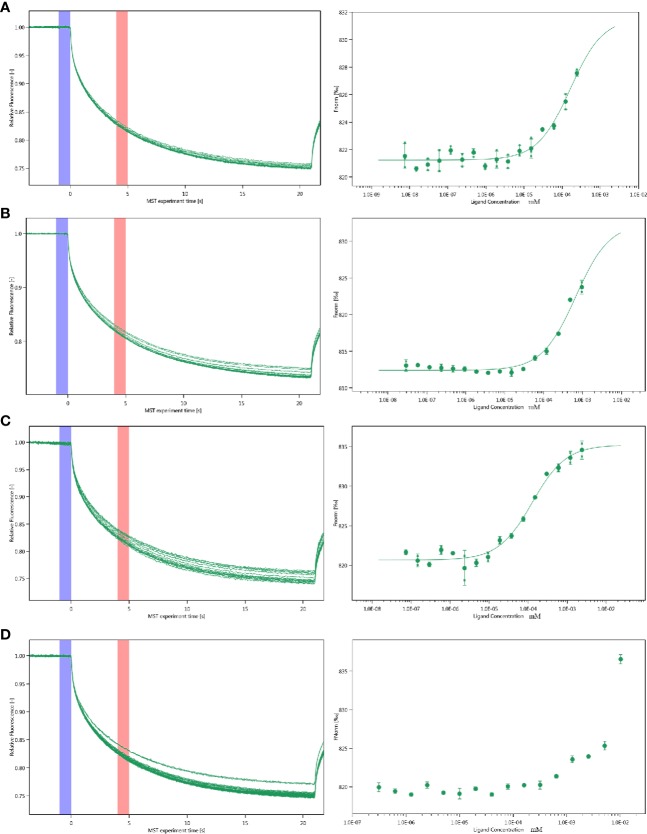
Molecular interactions of esculetin by NT. 115 analysis. MST time traces of esculetin at 16 different concentrations, from 7.48 × 10^−6^ to 0.245 mM **(A)** PGK2 (5.32 µM), Kd=161.0 ± 63.28 nM **(B)** GPD2 (0.02 µM), Kd=666.14 ± 196.12 nM **(C)** GPI (0.5 µM), Kd=127.36 ± 22.36 nM **(D)** BSA (0.945 µM), failed, MST signal measured 30 s after heating turned on (n=3).

The interactions between inhibitor and proteins were assayed by the MST. Multiply all values produce a relative change per thousand fluorescence. The Kd value of PGK2, GPD2 and GPI are shown as **Figure 5** and the Kd of negative control BSA is failed.

### Binding of the Proteins With High Degrees

**Figure 6:**
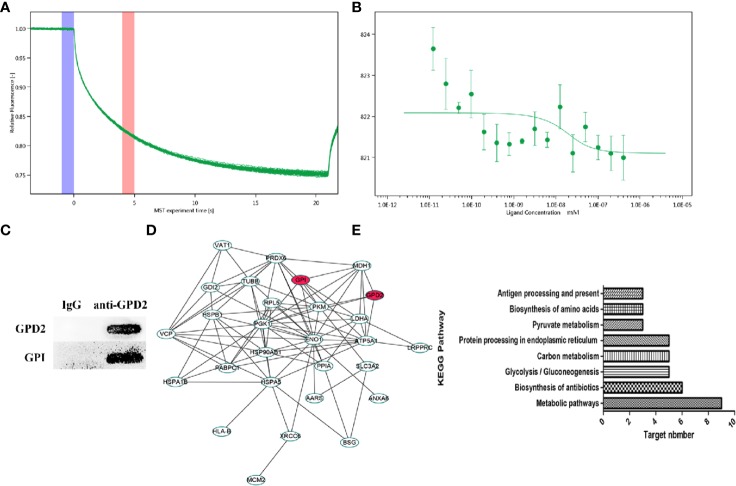
**(A**, **B)** MST time traces of 16 different GPI concentrations (1.23 × 10^−5^ to 0.403 µM) with a fixed concentration of GPD2 (0.02 µM), Kd = 8.59 ± 32.05 nM. MST signal measured 30 s after heating turned on (n=3). **(C)** The coimmunoprecipitated proteins were detected by western blot. **(D)** PPI network of differentially proteins. **(E)** Cancer-related signaling pathways associated with differentially proteins.

Using network pharmacology, we screened GPD2 and GPI with the high degree of affinity. We further established the binding between protein combinations and calculated the Kd by MST. According to the association curve, proteins have a conformational change with a S-shaped (**Figures 6A, B**). The result of Co-IP also indicated that the proteins bind well to each other (**Figure 6C**) and the differentially expressed genes were concentrated in glycolysis pathways (**Figures 6D, E**) (for proteomics data, **Table S2**).

### Esculetin Inhibits Tumor Growth in the Mouse Xenograft Model

**Figure 7:**
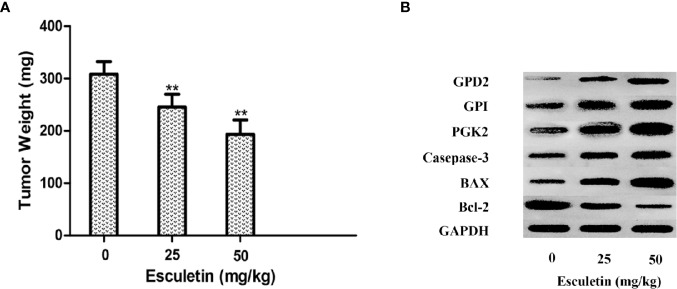
**(A)** Effects of esculetin on final tumor weight. **(B)** Comparison of PGK2, GPD2, GPI, Casepase-3, BAX, Bcl-2, and GAPDH levels in tumor tissue from different groups by WB, 0 mg/kg group was used as a control. Results are means± SEM, n=3 per experiment, *P < 0.05, **P < 0.01.

The antitumor activity of esculetin was evaluated by a xenograft of H22 cells in BALB/c mice. The tumor weight increased significantly in the model group compared with that in the esculetin treatment group (**Figure 7A**). Target protein measurement of the tumor tissue revealed an increase in PGK2, GPD2, and GPI in response to the esculetin treatment (**Figures 7B–D**). This may be a compensatory response when the proteins binding to small molecules were inhibited and thus the glycolysis-related process is blocked. The up-regulation of caspase-3, Bax, and the down-regulation of Bcl-2 indicated that the drug induced the apoptosis of tumor cells.

## Discussion

In this study, we have demonstrated that esculetin acts as a ligand for PGK2, GPD2, and GPI. The PGK2 is “open”: the N-terminal domain that can bind 3PG and the C-terminal domain that binds ATP are far apart to interact with the substrates (Blake and Evans, 1974; Watson et al., 1982; Bernstein et al., 1998; Flachner et al., 2004; Lee et al., 2006). Binding 3PG results in a 13-degree rotation that partially closes the structure and causes helix 13, which is disordered in the unliganded structure, to stabilize (Harlos et al., 1992; Davies et al., 1994; Auerbach et al., 1997; Bernstein et al., 1997; Szilágyi et al., 2001; Kovári et al., 2002). Esculetin has the same binding region as 3PG, and it can inhibit the activity of the enzyme.

With NAD as a cosubstrate, the GPD2 catalyzes the conversion of dihydroxy acetone phosphate (DHAP) and glycerol-3-phosphate (G3P). In the blood, GPDH is particularly important because it is responsible for maintaining the NAD/NADH balance in the glycosome through the oxidation of the NADH produced by glyceraldehyde-3-phosphate dehydrogenase during glycolysis. And GPI can promote cell proliferation and metastasis. Through binding to the two proteins, esculetin can inhibit their activity and control the energy metabolism of the tumor.

MST is very sensitive and can accurately quantify the interactions between molecules (Jerabek-Willemsen et al., 2014). The MST results suggest that the selected compound has potential molecular interactions with PGK2, GPD2, and GPI. According to Seidel et al. (Seidel et al., 2013), the fitted curve can be S-shaped or its mirror surface. The standard symbol for MST amplitude bases on the binding site of the compound and the conformational changes. Compounds with a positive slope are important for conformational change. Then, the anti-cancer effect may contain attenuating the conformational changes required for PGK2, GPD2, and GPI activation.

GO enrichment found that targets of the proteomics results are located in the extracellular exosome, membrane, cytosol, and other cell compartments. Then, the targets involved protein binding, poly(A) RNA binding, and ATP binding, and they were related to gluconeogenesis and canonical glycolysis. The drug may treat cancer by binding to PGK2, GPD2, and GPI and inhibit their energy metabolism and activity. The results of pathway enrichment also indicate that esculetin may play an anticancer role by inhibiting three targets.

The involvement of GPD2-GPI pathway has not been reported before, however, the role of both proteins in tumors has been repeatedly reported. Based on the proteomics and MST results, we get the Kd of the two proteins. The direct relationship between proteins and cancer will be studied later through gene editing techniques. Based on our results, we have found new targets for esculetin and the interaction between two proteins for the first time.

In recent years, with the deepening of studies on glucose metabolism of tumors, it has been found that tumor cells are accompanied by abnormal metabolism in addition to gene mutations and abnormal signaling pathways (Camarda et al., 2017; Ganapathy-Kanniappan, 2017; Wang and Lei, 2017). And, the abnormal metabolism of tumor cells exists in the whole process of tumor development, and the metabolic mode of tumor is different at each stage (Feng and Levine, 2010; Basetti, 2017). Wang et al. (Wang et al., 2013) compared the metabolomic of tumor tissues of patients with low malignant liver cancer and patients with high malignant liver cancer. The levels of lactic acid, glutamic acid, glutamine, glycine, leucine, alanine, and cholic acid in tumor tissues of patients with high malignant liver cancer were higher than those of patients with low malignant liver cancer and the levels of phosphatidylcholine, glycerol phosphatidylcholine, triglyceride, glucose, and glycogen were significantly lower than those in patients with low malignant liver cancer, which was consistent with Warburg effect. Based on the differences between different liver cancer cell lines, we will systematically study whether there are any potential differences in gene expressions and protein correlations after the incubation of esculetin in different cell lines, so as to provide a basis for future clinical application.

Esculetin exhibited characteristics typical of an allosteric inhibitor, and exerted its inhibitory effect by altering the conformation of PGK2, GPD2, and GPI. The binding ability of esculetin to protein was not strong enough, and the effect on protein activity may be weak at the same time. Then, it was possible that the compound also affected other biological processes, affecting the inhibition of proteins. However, it can provide the base for pharmacological research of other similar structure components in this herbal medicine and developing glycolytic-related antitumor drugs. These targets and the interaction between them also provide a new direction for their mechanism research.

## Data Availability Statement

The datasets generated for this study can be found in ProteomeXchange, Accession No. PXD017819. GEO, Accession No. GSE143274.

## Ethics Statement

The animal study was reviewed and approved by the Animal Care and Use Committee of Institute of Materia Medica, People’s Republic of China.

## Author Contributions

H-ZW conceived the study. H-ZW and Y-FY designed the study. S-TW performed the experiments. S-TW performed the data analysis. S-TW wrote the manuscript. BL, Z-ZA, P-TY, and Z-CH revised the manuscript.

## Funding

This work was supported by the National Natural Science Foundation of China, grant number No. 31570343.

## Conflict of Interest

The authors declare that the research was conducted in the absence of any commercial or financial relationships that could be construed as a potential conflict of interest.
