# Supplementary material for: Esculetin Inhibits Cancer Cell Glycolysis by Binding Tumor PGK2, GPD2, and GPI
Source: Front Pharmacol. 2020 Mar 27;11:379. doi: 10.3389/fphar.2020.00379 (PMC7118906; doi:10.3389/fphar.2020.00379)
Supplement: Supplementary file 1 [file Table_1.docx]

**TABLE S1.** The top different genes between the esculetin group and blank group.

| Gene name | Gene description |
| --- | --- |
| PGM2L1 | phosphoglucomutase 2 like 1 |
| TPI1P2 | triosephosphate isomerase 1 pseudogene 2 |
| AC002996.1 | novel protein |
| C5orf17 | chromosome 5 open reading frame 17 (putative) |
| PFKFB2 | 6-phosphofructo-2-kinase/fructose-2,6-biphosphatase 2 |
| ALDH1A3 | aldehyde dehydrogenase 1 family member A3 |
| DLAT | dihydrolipoamide S-acetyltransferase |
| ACSS2 | acyl-CoA synthetase short chain family member 2 |
| LDHA | lactate dehydrogenase A |
| PKM | pyruvate kinase M1/2 |
| ALDOC | aldolase, fructose-bisphosphate C |
| PGM2 | phosphoglucomutase 2 |
| PDHA1 | pyruvate dehydrogenase E1 alpha 1 subunit |
| PFKL | phosphofructokinase, liver type |
| PGK1 | phosphoglycerate kinase 1 |
| TMEM147-AS1 | TMEM147 antisense RNA 1 |
| DLD | dihydrolipoamide dehydrogenase |
| ALDH7A1 | aldehyde dehydrogenase 7 family member A1 |
| GPI | glucose-6-phosphate isomerase |
| PGAM1 | phosphoglycerate mutase 1 |
| TPI1 | triosephosphate isomerase 1 |
| ADH5 | alcohol dehydrogenase 5 (class III), chi polypeptide |
| PDHB | pyruvate dehydrogenase E1 beta subunit |
| PFKM | phosphofructokinase, muscle |
| ADPGK | ADP dependent glucokinase |
| HK2 | hexokinase 2 |
| AC093512.2 | Homo sapiens uncharaterized LOC112694756 (LOC112694756), transcript variant 1, mRNA. |
| ALDH3B1 | d |
| PFKP | phosphofructokinase, platelet |
| ALDOA | aldolase, fructose-bisphosphate A |
| GAPDH | glyceraldehyde-3-phosphate dehydrogenase |
| ENO2 | enolase 2 |
| G6PC3 | glucose-6-phosphatase catalytic subunit 3 |
| ALDH1B1 | aldehyde dehydrogenase 1 family member B1 |
| AC006064.4 | novel transcript, antisense to GAPDH |
| PCK2 | phosphoenolpyruvate carboxykinase 2, mitochondrial |
| PGM1 | phosphoglucomutase 1 |
| HK1 | hexokinase 1 |
| ALDH2 | aldehyde dehydrogenase 2 family member |
| ACSS1 | acyl-CoA synthetase short chain family member 1 |
| GAPDHP35 | glyceraldehyde 3 phosphate dehydrogenase pseudogene 35 |
| PFKFB1 | 6-phosphofructo-2-kinase/fructose-2,6-biphosphatase 1 |
