# Supplementary material for: Esculetin Inhibits Cancer Cell Glycolysis by Binding Tumor PGK2, GPD2, and GPI
Source: Front Pharmacol. 2020 Mar 27;11:379. doi: 10.3389/fphar.2020.00379 (PMC7118906; doi:10.3389/fphar.2020.00379)
Supplement: Supplementary file 2 [file Table_2.docx]

**TABLE S2.** The top different 50 proteins between the esculetin group and blank group.

| **N** | **unused** | **Acc** |
| --- | --- | --- |
| 1 | 14.05 | sp\|P43304\|GPDM_HUMAN |
| 2 | 13.3 | sp\|P16403\|H12_HUMAN |
| 3 | 13.11 | sp\|P18206\|VINC_HUMAN |
| 4 | 13.05 | sp\|P49748\|ACADV_HUMAN |
| 5 | 12.89 | sp\|Q01650\|LAT1_HUMAN |
| 6 | 12.41 | sp\|P53396\|ACLY_HUMAN |
| 7 | 12.04 | sp\|P80723\|BASP1_HUMAN |
| 8 | 11.98 | sp\|P00338\|LDHA_HUMAN |
| 9 | 11.87 | sp\|Q06830\|PRDX1_HUMAN |
| 10 | 11.77 | sp\|P11142\|HSP7C_HUMAN |
| 11 | 11.21 | sp\|P06744\|G6PI_HUMAN |
| 12 | 9.71 | sp\|P32119\|PRDX2_HUMAN |
| 13 | 9.59 | sp\|P04075\|ALDOA_HUMAN |
| 14 | 9.16 | sp\|P14625\|ENPL_HUMAN |
| 15 | 9.03 | sp\|P62987\|RL40_HUMAN |
| 16 | 8.83 | sp\|P32004\|L1CAM_HUMAN |
| 17 | 8.75 | sp\|P07437\|TBB5_HUMAN |
| 18 | 8.47 | sp\|P46940\|IQGA1_HUMAN |
| 19 | 8.31 | sp\|P21796\|VDAC1_HUMAN |
| 20 | 8.23 | sp\|P60174\|TPIS_HUMAN |
| 21 | 7.76 | sp\|P37802\|TAGL2_HUMAN |
| 22 | 7.46 | sp\|Q14974\|IMB1_HUMAN |
| 23 | 7.38 | sp\|P62937\|PPIA_HUMAN |
| 24 | 7.18 | sp\|P08174\|DAF_HUMAN |
| 25 | 7.11 | sp\|P51148\|RAB5C_HUMAN |
| 26 | 7.09 | sp\|P45880\|VDAC2_HUMAN |
| 27 | 6.92 | sp\|P04792\|HSPB1_HUMAN |
| 28 | 6.9 | sp\|P46977\|STT3A_HUMAN |
| 29 | 6.85 | sp\|P25705\|ATPA_HUMAN |
| 30 | 6.83 | sp\|P06733\|ENOA_HUMAN |
| 31 | 6.58 | sp\|P55072\|TERA_HUMAN |
| 32 | 6.45 | sp\|P09923\|PPBI_HUMAN |
| 33 | 6.13 | sp\|Q92945\|FUBP2_HUMAN |
| 34 | 6.13 | sp\|P10809\|CH60_HUMAN |
| 35 | 6.04 | sp\|Q00765\|REEP5_HUMAN |
| 36 | 6 | sp\|P62805\|H4_HUMAN |
| 37 | 5.82 | sp\|P00390\|GSHR_HUMAN |
| 38 | 5.69 | sp\|Q9UBG0\|MRC2_HUMAN |
| 39 | 5.65 | sp\|Q5VTE0\|EF1A3_HUMAN |
| 40 | 5.62 | sp\|Q96KK5\|H2A1H_HUMAN |
| 41 | 5.51 | sp\|Q99879\|H2B1M_HUMAN |
| 42 | 5.49 | sp\|P21333\|FLNA_HUMAN |
| 43 | 5.39 | sp\|O96005\|CLPT1_HUMAN |
| 44 | 5.33 | sp\|P07900\|HS90A_HUMAN |
| 45 | 5.31 | sp\|P43121\|MUC18_HUMAN |
| 46 | 5.27 | sp\|P11021\|GRP78_HUMAN |
| 47 | 5.21 | sp\|P07954\|FUMH_HUMAN |
| 48 | 5.18 | sp\|O75369\|FLNB_HUMAN |
| 49 | 5.18 | sp\|Q92928\|RAB1C_HUMAN |
| 50 | 4.97 | sp\|P07910\|HNRPC_HUMAN |
